# The biological effect of a beef‐derived *Latilactobacillus sakei* on beef steaks during chilled storage

**DOI:** 10.1002/fsn3.3143

**Published:** 2022-11-14

**Authors:** Huixuan Yang, Xin Luo, Lixian Zhu, Rongrong Liang, Yanwei Mao, Xiaoyin Yang, Lebao Niu, Yimin Zhang, Pengcheng Dong

**Affiliations:** ^1^ Lab of Beef Processing and Quality Control, College of Food Science and Engineering Shandong Agricultural University Tai'an China; ^2^ National R&D Center for Beef Processing Technology Tai'an China

**Keywords:** beef‐derived *L. sakei*, beef spoilage, lactic acid bacteria, microbial diversity, *Pseudomonas* spp

## Abstract

The aim of this study was to investigate the biological inhibiting effect of a beef‐derived *Latilactobacillus sakei* (RS‐25) on the spoilage of beef steaks in overwrapped packaging during the 12 days of storage at 4°C. Beef quality as well as microbial indicators were determined at different intervals during the storage after the inoculation of RS‐25 at the 6 log CFU/g, and the high‐throughput sequencing was applied to investigate the changes of microbial community structure during the storage. The inoculation of RS‐25 on beef had no effect (*p* > .05) on pH, TBARS, and TVB‐N during storage indicating the weak effect of such strain on the eat quality. Furthermore, the rise of L* and the delayed decline of a* and b* reveal the protection effect of RS‐25 on the meat color. RS‐25 reduced the re‐contaminated *Salmonella typhimurium* by 1.16 log CFU/g (*p* < .01), and the growth of *Brochothrix thermosphacta* was also inhibited but no inhibition was found on the *Pseudomonas* spp. at the first 6 days of storage. The inhibiting effect of RS‐25 was covered by the rapid growth of other microorganism during the following 6 days of storage. Consistent with the microbial counts results, high‐throughput sequencing analysis confirmed that the inoculated *L. sakei* RS‐25 was dominant at first 6 days, and then replaced by *Pseudomonas* spp. The findings obtained from the current study may provide basic information for the further application of bioprotective bacteria in preservation of beef steaks in the overwrapped packaging.

## Graphical abstract

Biological inhibiting effect of a beef‐derived *Latilactobacillus sakei* on the spoilage of steaks was investigated. The effect of LAB on the microflora structure of overwrapped packaging was investigated by sequencing analysis. Inoculation of LAB at 6 log CFU/g had a limited inhibitory effect on the dominant *Pseudomonas* during storage.

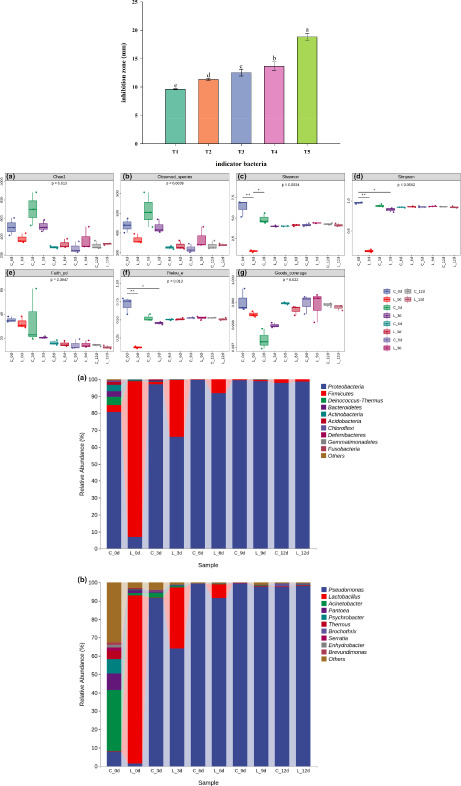

## 1 INTRODUCTION

Because of the high nutrient and moisture content, fresh meat is easy to be contaminated by microorganisms, which leads to 21% of total food losses (Jayasena & Jo, 2013; Shao et al., 2021; Slima et al., 2017). Furthermore, bacterial foodborne diseases were major food safety issue, and there is no “single intervention‐single chain point” combination that can completely eliminate the pathogens from the entire beef production chain (Buncic et al., 2014). Therefore, effective antibacterial strategies are needed to inhibit or reduce the survival of microorganisms in fresh beef. Since modern consumers are more inclined to high‐quality, healthy, safe, minimally processed, and preservative‐free foods (Castellano et al., 2017), lactic acid bacteria (LAB) have been received extensive attention as a natural biological antibacterial agent (Favaro & Todorov, 2017).

Lactic acid bacteria have a long history of safe use and have the qualified presumption of safety (QPS) as determined by the European Food Safety Authority (EFSA) (Koutsoumanis et al., 2020). At present, more than 60 strains of LAB have been recognized by the US Food and Drug Administration (FDA) as generally recognized as safe (GRAS). LAB are widely used in fermented meat products as starter cultures, and can also be added to meat products as protective cultures without destroying the original quality of the meat products (Barcenilla et al., 2022; Lücke, 1994). For the use in meat products, bioprotective bacteria showed good bacteriostasis effect in sausages (Castilho et al., 2020; Giello et al., 2018; Slima et al., 2017), patties (Castellano et al., 2011; Comi et al., 2015) and ham (Danielski et al., 2020; Hequet et al., 2007), and no negative effects on the shelf life of those products were found. Chen et al. (2020) found that the degree of dominance of *Lactobacillus* spp. in the beef microflora was considered to be a key factor dictating the shelf‐life. Hequet et al. (2007) inoculated cooked ham with 6 log CFU/cm^2^ of *Latilactobacillus sakei*, and the *Listeria innocua* under the control of *L. sakei* did not grow significantly throughout the 60‐day storage period.

The ability to produce many antibacterial metabolites was the main reason why LAB are used as an antibacterial agent (Leyva Salas et al., 2017). Organic acids such as lactic and acetic acid are the main metabolites of LAB. The low pH environment created by such organic acids is an important factor in inhibiting the growth of undesirable microorganisms (Özcelik et al., 2016). Except organic acids, bacteriocins synthesized by ribosomes of LAB also had a wide range of antimicrobial activities and displayed a target specificity against many strains (Woraprayote et al., 2016). Other metabolites of LAB exhibiting antibacterial effects such as H_2_O_2_, reuterin, and exopolysaccharide were also investigated (Bajpai et al., 2015; Ito et al., 2003; Ortiz‐Rivera et al., 2017). Moreover, the competition mechanism exhibited by LAB is also an important antibacterial pathway. It was reported that when LAB was co‐cultured with *Escherichia coli* O157:H7, proteins related to glucose metabolism, energy production, and several other metabolic pathways were over‐expressed, and this accelerated their own metabolic rate and improved their ability to compete with other strains for nutritional substrates (Orihuel et al., 2018). This also revealed that LAB may have signal‐sensing systems to sense the presence of other microorganisms and respond to stress. Some metabolite of LAB can act as quorum‐sensing inhibitors, reducing the release of N‐acyl homoserine lactone (AHL) signaling molecules that mediate quorum sensing, thereby inhibiting the formation of pathogenic microbial biofilms (Rana et al., 2020).

Although many factors have the potential capacity to inhibit other microorganism in theory, when it comes to the application, the practical effect depends on many factors such as pH, salt concentration, diffusible water phase, lipid and proteases in meat, and the interactions with other food ingredients or members of the food microbiome (Barcenilla et al., 2022; Blom et al., 1997). The interaction of bacteriocins with lipids in meat, or their inactivation by proteases, will lead to decreased antibacterial ability (Castilho et al., 2020; Favaro & Todorov, 2017). For example, *Enterococcus faecalis* B1, an antibacterial bacteriocin‐producing strain, did not reduce *L. monocytogenes* by itself in dry‐cured ham (Montiel et al., 2019). Moreover, there are often significant application differences between bioprotective strains. In the study of Iacumin et al. (2021) four bioprotective strains inoculated on cold‐smoked sea bass at 5 log CFU/g, and only *L. sakei* LAK‐23 successfully controlled the growth of *L. monocytogenes* when the storage reached 60 d. Therefore, it is necessary to evaluate the actual application effect of bioprotective candidate strains in meat.

Overwrapped packaging is one of the common meat packaging methods, but the simple packaging leads to a short shelf life. It is particularly important to apply additional food hurdle technology to improve the shelf life of the meat products in this packaging form. The inhibitory effect of LAB on pathogenic bacteria and spoilage bacteria in meat has been preliminarily explored in an aerobic environment and the application potential has been verified. Maragkoudakis et al. (2009) demonstrated that LAB retarded the growth of *L. monocytogenes* and *Salmonella enteritidis* in ground chicken within 7 days of storage. Under the same storage condition, Morales et al. (2020) also found the reduced number of *Pseudomonas* by about 3 log CFU/g by high concentrations of *L. sakei* on the surface of fresh chicken. Vold et al. (2000) isolated different background flora (about 80% of *L. sakei*) from ground beef and re‐inoculated it into overwrapped ground beef, and found that a high concentration of microbial background effectively inhibited the growth of *E. coli* O157:H7. At the same time, Danielski et al. (2020) applied *Carnobacterium maltaromaticum* to overwrapped packaging ready‐to‐eat meat product, and found it significantly reduced the number of *L. monocytogenes* by more than 2 log CFU/g compared to the control group after 7 days of storage. Taken together, LAB have an application potential in overwrapped packaged meat products. As far as our knowledge, the application of bioprotective bacteria which isolated from fresh beef and the use of such strain in the following beef cuts preservation is still scarce, and the impact of incubating such strain on the changes of bacterial community structural in beef during the storage of meat needs to be further clarified.

The aim of this study was to clarify the biological protection ability of one beef‐derived LAB and its impact on the quality of fresh beef. The quality traits including pH, color, TBARS and TVB‐N, and microbial composition including *Pseudomonas*, *B. thermosphacta*, *Enterobacteriaceae*, LAB, and microbial diversity of overwrapped packaged fresh beef when re‐contaminated by 6 log CFU/g *L. sakei* RS‐25, were analyzed during 12 days of storage. The inhibitory ability of *L. sakei* RS‐25 against *S. typhimurium* during storage was also evaluated.

## 2 MATERIALS AND METHODS

### 2.1 Bacterial strains

The *L. sakei* RS‐25 strain (GenBank accession number: ON007101) was isolated from chilled beef previously in our laboratory. It was enriched for 24 h at 37°C in Man Rogosa & Sharpe (MRS) broth (Land Bridge Co., Beijing, China). As indicator bacteria to detect the antagonistic activity of *L. sakei*, *L. monocytogenes* serotype 4b ATCC 19115, *L. monocytogenes* serotype 1/2a CMCC 54004, *Escherichia coli* O157:H7 S2, *Staphylococcus aureus* ATCC 25923 and *S*. *typhimurium* ATCC 14028 were all strains preserved in our laboratory, and they were propagated at 37°C in Trypticase Soy Broth (TSB) (Land Bridge Technology Co., Beijing, China).

### 2.2 Antagonistic activity against pathogens

In this experiment, cell‐free supernatant (CFS) of *L. sakei* RS‐25 was used for in vitro anti‐pathogenic bacteria activity. Briefly, an overnight MRS broth of *L. sakei* RS‐25 was centrifuged at 8200 *g* for 20 min at 4°C and filtered through the millipore filter (0.22 μm) to collect the culture supernatants. Referring to Arrioja‐Bretón et al. (2020) the agar diffusion method was used to determine the inhibitory activity of LAB against pathogenic bacteria, and the final concentration of the indicator bacteria was 10^6^ CFU/ml. Oxford cup (8 mm) was cut in the solidified agar and filled with 240 μl of cell‐free supernatant. The diameter of each inhibition zone was measured using three parallel wells per group and repeated three times independently.

### 2.3 The preparation for the overwrap‐packaged beef

Four Simmental crossbred cattle of about 24 months were randomly selected in a commercial slaughterhouse (Yangxin, Shandong, China). After slaughter and chilling in accordance with the GB/T 19477‐2018 slaughter operating procedures, the M. *longissimus lumborum* of the left half carcasses (*n* = 4) were collected, and then transported to the laboratory under refrigerated conditions for further analysis. Each beef loin was cut into 12 steaks, and then each steak was aseptically cut into five 4 × 4 × 2 cm^3^ pieces, and finally all 60 steak pieces were randomly allocated into five storage points in four treatment groups.

In order to test the bioprotective characteristics of *L. sakei* RS‐25 and its effect on beef quality during 12 days of storage, beef samples were re‐contaminated with the LAB or sterile water. At the same time, since *L. sakei* showed the best bacteriostatic effect against *S. typhimurium* in vitro, it was selected as the indicator bacteria to further study in meat. For the re‐contamination, the cells of *L. sakei* RS‐25 were centrifuged at 8000 rpm for 20 min and washed twice with phosphate buffer solution before being collected for use (Slima et al., 2017). Besides control group (G0): No inoculation, sprayed with sterile water, three different treatments were applied as following: LAB treatment group (G1): Beef samples were sprayed with *L. sakei* RS‐25 at 6 log CFU/g; *S. typhimurium* Group (G2): Beef samples were sprayed with *S. typhimurium* at 3 log CFU/g; *S. typhimurium* and *L. sakei* group (G3): Beef samples were sprayed with *S. typhimurium* at 3 log CFU/g, dried in a biological safety cabinet for 10 min, and then inoculated with *L. sakei* RS‐25 to a final concentration of 6 log CFU/g. Among them, the quality and spoilage microbial indicators of the G0 and G1 were compared, and the bacterial number of G2 and G3 were counted to evaluate the ability of the RS‐25 strain to prevent and control *Salmonella* spp. in beef.

After the bacterial liquid was absorbed by the meat, the sample was transferred to the overwrapped package tray (TQBC‐0775, Oxygen transmission rate at 23°C was 20 cm^3^/m^2^/24 h/0% relative humidity [R.H.]; water vapor transmission rate at 38°C was 15 g/m^2^/24 h/90% R.H.) (Sealed Air, Shanghai, China), and covered with PE film. The beef samples of each treatment were randomly divided into five time points (0, 3, 6, 9, and 12 days), and quality and microbiological indicators were analyzed at each time point. All samples were stored at 4°C.

### 2.4 pH determination

The pH value of beef was measured with a pH probe (SenvenGo, Mettler‐Toledo, Im Langacher Greifensee, Switzerland) during storage on 0, 3, 6, 9, and 12 days. The pH probe, which was calibrated by buffers with pH 4.00 and 7.00 at room temperature, and the values were recorded on six locations randomly in each sample and the average values were calculated.

### 2.5 Color measurement

The surface color of steak cuts at 0 d was measured after blooming for 30 min exposed to the air at 0–4°C by using a spectrophotometer (Illuminant A, 4 mm diameter aperture, 10°observer, SP62, X‐Rite, Inc., Grand Rapids, USA). Steak cuts at 3, 6, 9, and 12 d were measured immediately after opening the package. Six areas were randomly selected from each cut to determine the lightness (L*), redness (a*), and yellowness (b*). Total color differences (Δ*E*) were calculated using the following equation: [(L*_1_–L*_0_)^2^ + (a*_1_–a*_0_)^2^ + (b*_1_–b*_0_)^2^]^1/2^, where the subscript 1 represents the LAB treatment group at different time points and the subscript 0 represents the control group.

### 2.6 Lipid oxidation

Lipid oxidation was assessed by measurement of TBARS using the method of Siu and Draper (1978) with some modification. Samples were taken from the surface of the steaks at each sampling day and stored at −80°C before analysis. Samples (1 g) were transferred to 4 ml of distilled water and ground with a freeze‐grinding instrument (Jingxin, Shanghai, China) for 1 min, and then 4 ml of 10% trichloroacetic acid (TCA) was added to the grinding solution. After filtering the grinding solution, 1 ml of the filtrate was taken and 0.25 ml of 0.06 M 2‐thiobarbituric acid (TBA) was added, and then incubated at 80°C for 90 min. After that, the absorbance (532 nm) was measured with a microplate reader. Results were expressed as mg malondialdehyde (MDA) equivalent per kg of beef.

### 2.7 Total volatile basic nitrogen (TVBN)

The concentration of TVBN was determined with reference to the Chinese National Food safety standard method GB 5009.228‐2016. A sub‐sample of the steaks was collected at each sampling day and stored at −20°C before analysis. Distilled water (75 ml) was added to ground samples (10 g), with shaking at room temperature and immersion for 30 min. After that, the homogenate was transferred to a distillation tube and 1 g of magnesium oxide was added, which was connected to the automatic Kjeldahl nitrogen determination apparatus (K ‐ 355, Buchi, Switzerland), and the volatile basic nitrogen was absorbed by the boric acid solution (20 g/L). Calculated as follows:

$$\text{TVBN}\;\left(\mathrm{mg}/100\;g\right)=\left(\left[\text{V1}-\text{V2}\right]\times c\times 14\right)/m\times 100.$$

Where: V1 denotes the volume of hydrochloric acid (ml) consumed by the tested sample. V2 denotes the volume of hydrochloric acid (ml) consumed by the blank sample. c denotes the actual concentration of hydrochloric acid (mol/L). m denotes the weight of the sample (g).

### 2.8 Microbial analysis

Beef samples (10 g) were collected aseptically from the surface of the steak, and then transferred to stomacher bags (BagPageR®; Interscience, St Nom, France) containing 90 ml of sterile normal saline (1% tryptone and 0.85% sodium chloride), and mixed in a blender (BagMixerR®400; Interscience, St Nom, France). The bacterial solution was serially diluted with 0.1% (w/v) sterile peptone water and the LAB, *B. thermosphacta*, *Enterobacteriaceae*, and *S. typhimurium* in beef were counted at each sampling day during the 12 days of chilled storage.

For the re‐inoculation experiment in beef, the cultivation environment of the various microorganisms was as follows: The number of LAB were cultured and counted on MRS medium at 37°C for 48 h. *B. thermosphacta* was selectively cultured and counted in *Steptomycin thallous* acetate agar (STAA) (Hope Bio Co., Qingdao, China) with corresponding culture media supplements after incubation at 25°C for 48 h. *Enterobacteriaceae* was screened and counted on violet red bile glucose agar (VRBGA) (Land Bridge Co., Beijing, China) after incubation at 37°C for 24 h. *S. typhimurium* was observed and counted on the *Salmonella* color culture medium (CHROMagar™, France).

### 2.9 DNA extraction and high‐throughput sequencing

Samples (10 g) were taken from the surface of each steak at each sampling day, and the total genomic DNA of the bacteria was extracted (Chen et al., 2020; Yang et al., 2018). Sixty milliliters of liquid from the homogenate in section 2.7 was aspirated, muscle fibers and other tissues were removed through filtration, and then the bacterial cells were collected by centrifugation (10 min, 10,000 rcf). Total genomic DNA was extracted according to magnetic beads method provided by Shannuo™ Bacterial DNA Kit (Tianjing Charme Co., Ltd., China). After DNA concentration and integrity were tested, the 16 S rRNA V3‐V4 hypervariable region primers were amplified using 338F (5′−ACTCCTACGGGAGGCAGCA−3′) and 806R (5′−GGACTACHVGGGTWTCTAAT−3′), and the amplified products were purified and quantified.

### 2.10 Statistical analyses

Two‐way analysis of variance (ANOVA) of pH, meat color, TBARS, TVB‐N, and microbial counts was carried out using a general linear model in SPSS 26.0 (IBM, USA). The treatment (recontamination or not), storage time and their interaction were fixed factors, and the replicates (loin, *n* = 4) were the random factor. The data of in vitro antagonistic activity and Δ*E* were analyzed by one‐way ANOVA analysis, in which the indicator bacteria species and storage time were fixed effects, respectively. The effects were compared by LSD method and the differences were considered significant when *p* < .05.

Community DNA fragments were paired‐end sequenced using the Illumina platform, all data were compared with the sliva database. Sequencing raw data were saved in FASTQ format (https://en.wikipedia.org/wiki/Fastq). Raw sequence data were denoised, spliced, and de‐chimerized by DADA2 method (Callahan et al., 2016) with the QIIME2 (2019.4). Based on seed protein sequence of the corresponding functional gene on the RDP website, the insertion and deletion errors in the nucleic acid sequence were corrected by FrameBot (v1.2) (https://github.com/rdpstaff/Framebot). Alpha diversity was used to identify bacterial community richness (Chao1 and Observed species indices), diversity (Shannon and Simpson indices), evenness (Pielou's evenness indices), and coverage (Good's coverage). Hierarchical cluster analysis with the unweighted pair‐group method with arithmetic means (UPGMA) was performed by the uclust function of the R language (version 4.1.0). A heatmap was generated based on the relative abundance of each microbial genus using R script.

## 3 RESULTS AND DISCUSSION

### 3.1 Antagonistic activity against pathogens

**FIGURE 1:**
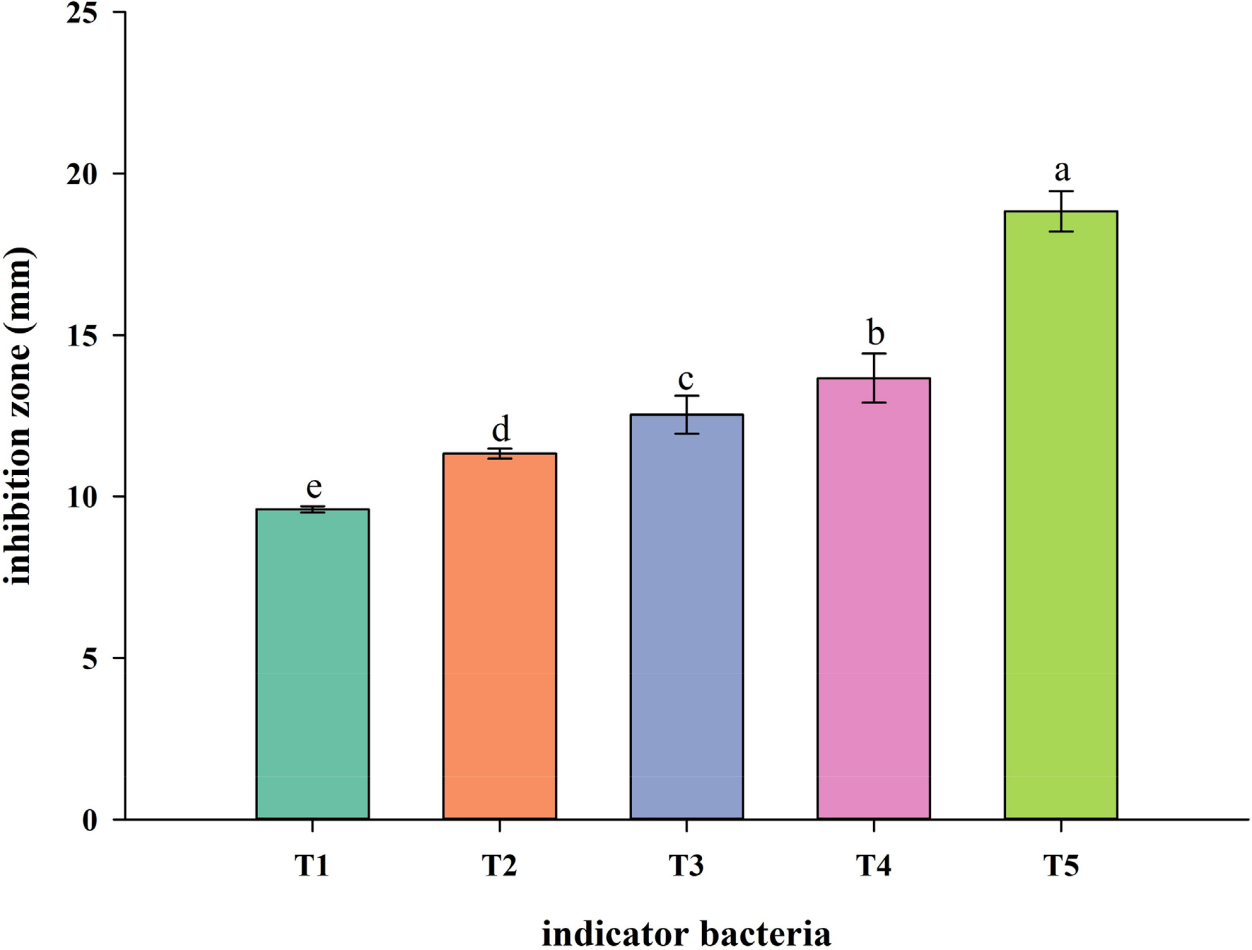
Antimicrobial activity of *L. sakei* RS‐25. A–E: Different letters showed significant difference among the diameter of inhibition zones (*P* < .05). T1: *L. monocytogenes* serotype 4b ATCC 19115; *L*. *monocytogenes* serotype 1/2a CMCC 54004; T3: *E. coil* O157:H7 S2; T4: *S*. *aureus* ATCC 25923; T5: *S. typhimurium* ATCC 14028

In this study, using *E. coli* O157:H7, *L. monocytogenes*, *S. typhimurium* and *S. aureus* as indicator strains, the in vitro antibacterial effect of *L. sakei* RS‐25 was determined. *L. sakei* RS‐25 has inhibitory effect on all indicator bacteria (inhibition zone > 8 mm), among which the antibacterial effect on *S*. *typhimurium* was the best, followed by *S. aureus*, and finally *E. coli* O157:H7 and *L. monocytogenes* (Figure 1). This was most likely related to the presence of organic acids in CFS. Decarboxylation systems such as glutamate decarboxylase (GAD) and arginine decarboxylase (ADI) systems exist in *E. coli* and *L. monocytogenes*, which consume intracellular hydrogen ions to maintain cellular pH homeostasis (Arcari et al., 2020). However, *S*. *typhimurium* lacks GAD system, so there are certain defects in acid resistance (Lin et al., 1995). We should note that the antagonism of LAB against pathogen also involves nutrient competition, and its actual antagonism is also affected by the meat matrix. Therefore, we selected *S*. *typhimurium* as the indicator bacteria to further explore the bioprotective effect of *L. sakei* RS‐25 in meat.

### 3.2 pH

The main effects of storage time, treatment, as well as their interaction had no effect on the pH value of beef samples (*p* > .05, Table 1). During the chilled storage period for 12 days, the pH value was maintained at the range of 5.51–5.57. This result agrees with the findings of Castellano et al. (2011), who found that after treatment with *L. curvatus* CRL705 and *Lactococcus lactis* CRL1109, the pH value of beef steaks did not change significantly over 9 days of storage. Another study conducted by Danielski et al. (2020) also reported that over 7 days after the ham surface was inoculated with *C. maltaromaticum*, no significant changes in pH value were found. However, a study reported that after a storage period of 38 days under vacuum conditions, *L. sakei* and *L. curvatus* significantly reduced the pH of fresh beef to 5.27 and 5.34, respectively (Zhang et al., 2018). This may be related to the accumulation of acid production due to longer storage time in the vacuum condition compared with the current study, and the fact that LAB can produce organic acids to reduce the pH. Jones et al. (2010) also found that the metabolites of *L. sakei* reduced the surface pH value of the lamb after 6 weeks. LAB are commonly used as a starter and added to fermented meat products, usually reducing the pH of the fermented meat to protect product safety. However, LAB acting as protective cultures (rather than starter cultures) should not alter the flavor and appearance of the product, especially the large drop in pH (Lücke, 1994).

**TABLE 1:** Effect of the re‐contamination of *L. sakei* RS‐25 on the color, pH, TBARS, TVB‐N of beef during chilled storage up to 12 days

	Treatment	Storage time (d)					Mean ± SE	*p*‐Value		
		0	3	6	9	12		Storage time	LAB treatment	Storage time * LAB treatment
L*	G1	43.9 ± 1.0	41.9 ± 0.8	39.1 ± 1.8	36.1 ± 1.4	30.4 ± 0.8	38.3 ± 0.3^x^	<.001	.025	.175
	G0	43.4 ± 1.0	41.8 ± 1.2	37.5 ± 1.8	33.4 ± 2.3	30.2 ± 3.2	37.3 ± 0.3^y^			
	Mean ± SE	43.6 ± 0.5^a^	41.8 ± 0.5^b^	37.5 ± 0.5^c^	35.6 ± 0.5^d^	30.3 ± 0.5 ^e^				
a*	G1	22.4 ± 1.4^xa^	19.6 ± 1.1^xb^	19.5 ± 1.4^xb^	19.0 ± 1.8^xb^	14.9 ± 1.9^xc^	19.0 ± 0.3	<.001	.136	<.001
	G0	23.6 ± 1.8^xa^	20.3 ± 1.3^xb^	19.0 ± 1.9^xbc^	18.0 ± 0.9^xc^	12.0 ± 1.0^yd^	18.6 ± 0.3			
	Mean ± SE	23.0 ± 0.4	19.7 ± 0.4	19.3 ± 0.4	18.6 ± 0.4	13.4 ± 0.4				
b*	G1	18.4 ± 1.4^xa^	16.9 ± 1.0^xb^	16.9 ± 1.0^xb^	16.2 ± 0.7^xb^	11.5 ± 1.8^xc^	16.0 ± 0.2	.016	<.001	.006
	G0	18.9 ± 1.3^xa^	17.7 ± 1.0^xa^	15.4 ± 1.3^xb^	14.9 ± 0.7^yb^	9.7 ± 1.7^yc^	15.3 ± 0.2			
	Mean ± SE	18.6 ± 0.3	17.3 ± 0.3	16.08 ± 0.32	15.6 ± 0.3	10.6 ± 0.3				
Δ*E*	Mean ± SE	1.46 ± 0.58^cd^	2.11 ± 0.44^c^	3.07 ± 1.04^b^	2.50 ± 0.47^bc^	4.32 ± 0.99^a^		<.001		
pH	G1	5.56 ± 0.03	5.55 ± 0.06	5.51 ± 0.06	5.52 ± 0.06	5.52 ± 0.07	5.53 ± 0.01^x^	.393	.111	.085
	G0	5.54 ± 0.04	5.55 ± 0.05	5.57 ± 0.09	5.56 ± 0.08	5.52 ± 0.10	5.55 ± 0.01^x^			
	Mean ± SE	5.55 ± 0.01^a^	5.55 ± 0.01^a^	5.54 ± 0.01^a^	5.54 ± 0.01^a^	5.52 ± 0.01^a^				
TBARS	G1	0.10 ± 0.04	0.11 ± 0.03	0.13 ± 0.01	0.19 ± 0.04	0.48 ± 0.08	0.21 ± 0.14^x^	<.001	.670	.529
	G0	0.08 ± 0.02	0.08 ± 0.02	0.11 ± 0.04	0.22 ± 0.02	0.55 ± 0.03	0.20 ± 0.13^x^			
	Mean ± SE	0.09 ± 0.02^c^	0.09 ± 0.02^c^	0.13 ± 0.02^c^	0.20 ± 0.02^b^	0.52 ± 0.03^a^				
TVB‐N	G1	9.12 ± 1.77	11.29 ± 1.32	11.34 ± 1.69	14.03 ± 2.22	17.61 ± 1.79	12.68 ± 0.46^x^	<.001	.340	.873
	G0	8.77 ± 1.40	10.76 ± 2.25	11.69 ± 0.53	13.29 ± 1.86	15.77 ± 1.28	12.05 ± 0.45^x^			
	Mean ± SE	8.95 ± 0.69^d^	11.02 ± 0.69^c^	11.51 ± 0.69^bc^	13.66 ± 0.79^b^	16.69 ± 0.74^a^				

*Note*: Control group ‐ No inoculation treatment (G0); LAB treatment group ‐ Beef samples were inoculated with *L. sakei* RS‐25 by 6 log CFU/g (G1); ^a‐e^Means with different letters under different storage time are significantly different (*p* < 0.05), ^x–y^Means with different letters under different treatment are significantly different (*p* < 0.05).

### 3.3 Meat color

The effect of the re‐contamination of *L. sakei* RS‐25 on the color of beef during chilled storage is shown in Table 1. The interaction between storage time and LAB treatment influenced the a* and b* values of the beef steaks (*p* < .05). The initial values of redness for the two groups did not show a significant difference. However, during the storage period from 0 to 12 days, the a* values of the LAB treatment group were more stable with the final values being 14.9 and 12.0, respectively, after 12 days of storage. The b* values showed the same trend as the redness, and the main effect of treatment also showed that after the re‐contamination with *L. sakei* RS‐25, the lightness and yellowness both increased compared with no‐contaminated samples. Yang et al. (2020) found that the presence of *Lactobacillus* spp. and *Lactococcus* spp. improved the redness and chroma, respectively, and decreased the methemoglobin ratio. Also, in the overwrapped packaging that was stored at 4°C for 12 days, Li et al. (2016) also observed the effect of LAB on delaying deterioration of meat color, even if the growth rate of aerobic bacteria exceeded that of LAB at the later stage, and believed that the increase in a* values in beef patties was related to the formation of red myoglobin derivatives. In this study, we observed that *L. sakei* RS‐25 increased the L* values and delayed the decline of a* and b* values of beef steaks, which means after being treated with *L. sakei* RS‐25, the beef color was improved. The total color difference (Δ*E*) value also demonstrated the retarding effect of LAB on the deterioration of color, where the higher Δ*E* value, the greater difference between the two samples. During the whole storage period, Δ*E* showed a significant (*p* < .05) increase trend with storage time. On 9 d of storage, the L* values decreased in both groups due to meat spoilage, resulting in Δ*E* from 3.07 to 2.50. At the end of storage (12 d), Δ*E* reached 4.32 due to a slower decline in a* and b* values in the LAB treatment group, which showed a greater color difference.

### 3.4 Lipid oxidation (TBARS) and TVB‐N

The detection of TBA is widely used in the evaluation of lipid oxidation in meat products, and TBARS value is one of the important indicators reflecting the quality of beef (Sun et al., 2001). Despite of incubating *L. sakei* RS‐25 or not, the storage time escalated the degree of lipid oxidation of beef samples (*p* < .05; Table 1). However, one noticeable point in this study is that the re‐contamination of *L. sakei* (6 log CFU/g) on the beef surface did not increase the TBARS values than the control in each sampling day. This indicated that the inoculated LAB did not aggravate the lipid oxidation during beef storage, which is consistent with the reports of Slima et al. (2017), who found that large number LAB (7 or 8 log CFU/g) as protective cultures did not aggravate lipid oxidation in fresh beef sausages.

A similar trend that the storage time increased the TVB‐N values despite of incubating *L. sakei* RS‐25 or not was also investigated (*p* < .05; Table 1). On the 9th day of storage, the control group and the LAB treatment group reached 13.3 mg/100 g and 14.0 mg/100 g, respectively, which were close to the spoilage limit standard value of fresh meat according to the National Food Safety Standard of China (GB 2707‐2016). Consistent with the results of lipid oxidation, the inoculation of *L. sakei* RS‐25 on the beef surface did not affect (*p* > .05) TVB‐N values in each sampling day (Table 1). These results were in accordance with the findings previously reported by Zhang et al. (2018) showing that after the steak surface was treated with 7 log CFU/g of *L. sakei* or *L. curvatus*, no increase in TVB‐N values was observed during storage for 4 weeks. In general, LAB have a minimal impact on meat quality, suggesting that even its large presence does not lead an acceleration of the lipid oxidation or meat spoilage.

### 3.5 Microbial counts

Table 2 shows the effect of inoculating *L. sakei* RS‐25 on the reduction of the bacteria in beef steak. The inherent number of LAB in the control group was 4.13 log CFU/g, which was higher than the two abattoirs in China investigated by Chen et al. (2020). In the G1 treatment group, after being inoculated with *L. sakei* RS‐25, the number of LAB reached 6 log CFU/g. Although *L. sakei* RS‐25 colonized in beef steaks successfully, no significant growth occurred during the first 6 days. At the end of storage (12 days), the number of LAB in the G1 G0 groups increased to 7.28 log CFU/g and 5.09 log CFU/g, respectively. Compared with the control group, *L. sakei* RS‐25 significantly reduced a certain number of spoilage bacteria within 6 days (Table 2), for instance, the population of *B. thermosphacta* was reduced 0.38 log CFU/g by 6 d of storage and *S. typhimurium* was also reduced 0.59 log CFU/g and 1.16 log CFU/g after 3 and 6 d of storage, respectively, for re‐contaminated steaks (Table 2, *p* < .05). Furthermore, the number of *S. typhimurium* did not increase (*p* > .05) within the first 6 days of storage compared to the control group (Table 2). There was no significant difference in the colony count of *Pseudomonas* between the two groups for the first 6 days of storage which indicating LAB had no inhibitory effect on *Pseudomonas* during such a storage period.

**TABLE 2:** Effect of the re‐contamination of lactic acid bacteria on the microbial loads in beef during chilled storage up to 12 days

Time (d)	*Enterobacteriaceae* (log CFU/g)		*Brochothrix thermosphacta* (log CFU/g)		*Pseudomonas* spp. (log CFU/g)		Lactic acid bacteria (log CFU/g)		*Salmonella typhimurium* (log CFU/g)	
	G0	G1	G0	G1	G0	G1	G0	G1	G2	G3
0	3.44 ± 0.11^ex^	3.23 ± 0.17^ey^	BDL	BDL	2.75 ± 0.26^ex^	2.80 ± 0.27^ex^	4.13 ± 0.26^dy^	6.02 ± 0.07^cx^	3.14 ± 0.64^dx^	2.68 ± 0.47^cy^
3	3.96 ± 0.19^dx^	3.68 ± 0.14^dy^	2.97 ± 0.47^dx^	2.87 ± 0.37^dx^	3.90 ± 0.51^dx^	3.75 ± 0.35^dx^	3.22 ± 0.43^cy^	6.07 ± 0.06^cx^	3.14 ± 0.56^dx^	2.55 ± 0.22^cy^
6	4.72 ± 0.17^cx^	4.66 ± 0.17^cx^	4.52 ± 0.33^cx^	4.14 ± 0.41^cy^	4.85 ± 0.42^cx^	4.69 ± 0.30^cx^	3.08 ± 0.50^cy^	6.17 ± 0.08^bcx^	3.99 ± 0.07^cx^	2.83 ± 0.35^cy^
9	6.52 ± 0.26^bx^	6.10 ± 0.18^by^	5.68 ± 0.54^by^	6.17 ± 0.29^bx^	6.56 ± 0.32^by^	7.04 ± 0.29^bx^	4.05 ± 0.15^by^	6.39 ± 0.31^bx^	6.33 ± 0.34^bx^	5.56 ± 0.62^by^
12	8.07 ± 0.21^ax^	8.06 ± 0.18^ax^	7.69 ± 0.65^ax^	7.87 ± 0.34^ax^	8.52 ± 0.27^ax^	8.80 ± 0.19^ax^	5.09 ± 0.01^ay^	7.28 ± 0.37^ax^	8.57 ± 0.54^ax^	8.30 ± 0.97^ax^

*Note*: Control group ‐ No inoculation treatment (G0); LAB treatment group ‐ Beef samples were inoculated with *L. sakei* RS‐25 at 6 log CFU/g (G1); *S. typhimurium* Group ‐ Beef samples were inoculated with *S. typhimurium* at 3 log CFU/g (G2); *S. typhimurium* and *L. sakei* group ‐ Beef samples were inoculated with *L. sakei* RS‐25 at 6 log CFU/g and *S. typhimurium* at 3 log CFU/g (G3). BDL, Below detection limit; ^a–e^Different letters indicate significant differences in different storage times of the same microbial index (*p* < .05); ^x–y^Different letters indicate significant differences in different treatments of the same microbial index (*p* < .05).

The inhibitory effect of *L. sakei* RS‐25 on *B. thermosphacta* and *S*. *typhimurium* may be the result of antagonistic effect among *L. sakei* and other autochthonous bacteria in raw meat such as the competition for nutrients, oxygen or hydrogen sources (Nychas et al., 2008). Effective utilization of nutrients such as glucose and glutamine by LAB can control the growth of spoilage bacteria (Honoré et al., 2016). Under the growth conditions of this study, the nutrient competition ability produced by 6 log CFU/g of *L. sakei* may contribute an effective nutrient competition effect. This may consist of the assumption of the Jameson effect that the dominating microbiota quantitatively inhibits growth of the pathogen in the same way that they inhibit their own growth, and the pathogen therefore stops growing at the time when the dominating microbiota reach their maximum population density. Other studies also find the same phenomenon, whether the original beef microflora consisted mainly of LAB of which approximately 80% were *L. sakei* (Vold et al., 2000) or a single strain with bacteriostatic properties (Danielski et al., 2020) was used as a high‐concentration microbial background, its effective prevention and control of pathogenic microorganisms in overwrapped packaging was confirmed. Other reports also confirmed that high concentrations of microflora in raw meat inhibited the growth of *Salmonella* spp. (Moller et al., 2013; Zaher & Fujikawa, 2011).

*Latilactobacillus sakei* showed bioprotective potential within 6 days, however not as effective as applications in vacuum packaging. In fact, the other microorganisms grew significantly after 6 d of storage in this study (Table 2), at which point the inhibitory ability of LAB disappeared. LAB inoculated on vacuum‐packed steaks by Zhang et al. (2018) extended the shelf life of steaks by at least 10 days. Vold et al. (2000) compared the application effects of high‐concentration background microorganisms between vacuum and overwrapped packaging, and also found that *L. sakei* had bacteriostatic effects in both packaging situations, and the inhibition was more pronounced in vacuum packaging. As mentioned before, *L. sakei* RS‐25 did not significantly reduce the pH value in the current condition and this may weaken the inhibiting effect of LAB in a large extent. The inhibition of other bacterial species may be an advantage in the number of LAB. Morales et al. (2020) observed the inhibition of *Pseudomonas* by 8 log CFU/g LAB inoculum on chicken surfaces, which may be due to the stronger nutrient competition potential of higher initial LAB numbers. Other researchers argued that the ability of LAB to produce available metabolites is an important factor in their antibacterial properties (Ioannis et al., 2014). Bolívar et al. (2021) observed the inhibitory effect on *L. monocytogenes* when *L. sakei* reached the maximum population density, and they believed that bacteriocin may play a special antagonistic effect at this time. However, it may be difficult for LAB to metabolize sufficient levels of bacteriostatic substances in meat compared to in pure culture medium, and the presence of these bacteriostatic substances such as bacteriocins in meat has not been quantified in relevant studies (Bolívar et al., 2021; Costa et al., 2019). Morales et al. (2020) found that the antibacterial effect of *L. sakei* on *Pseudomonas* spp. in actual application on fresh chicken was far less than that in pure culture medium. Therefore, whether the inhibitory effect is due to metabolites or bacterial population remains to be further explored.

It was also found in the current study that the storage period of 6 d seems to be a critical time point (Table 2). After 6 days, the number of most spoilage bacteria had exceeded 6 log CFU/g, which was also the initial inoculum of *L. sakei*. It is worth noting that *B. thermosphacta*, *Pseudomonas* spp., and *S. typhimurium* had rapid growth after the 6th day of storage in the presence of lactic acid bacteria inoculation (G1), and the growth rate exceeded that of the control group (G0). This may be related to the synergistic effect of *Pseudomonas* spp. with other microorganisms. *Pseudomonas* have a strong ability to hydrolyze carbohydrate and lipids, which would made more readily available sources of carbohydrates. On the other hand, the hydrolysis of proteins by *Pseudomonas* can release peptides and amino acids (Marshalld & Schmidtr, 1991). The more readily available nutrients create favorable conditions for the growth of other spoilage and pathogenic bacteria, even LAB themselves.

Due to the lower spoilage ability of *L. sakei* RS‐25 in this study, and the 6 log CFU/g microbial background provided in the meat, the above data suggest that the microbial background has a certain protective effect in the first 6 days. However, *L. sakei* RS‐25 lost its dominant position in the later period, especially after the rapid growth of *Pseudomonas* spp. Increasing the number of microbial background or combining with other antibacterial substances (such as chitosan, fructooligosaccharides, etc.) may be a better method to deal with this situation, and subsequently, prolong the shelf life of overwrapped packaging meat. The plate counting method cannot accurately account for the dynamic changes of various microorganisms in the meat during the spoilage process. In order to deeply analyze the influence of *L. sakei* on the bacterial succession of beef microflora, the bacterial composition of each sample was further studied by high‐throughput sequencing.

### 3.6 Microbial diversity

#### 3.6.1 Sequencing analysis

**FIGURE 2:**
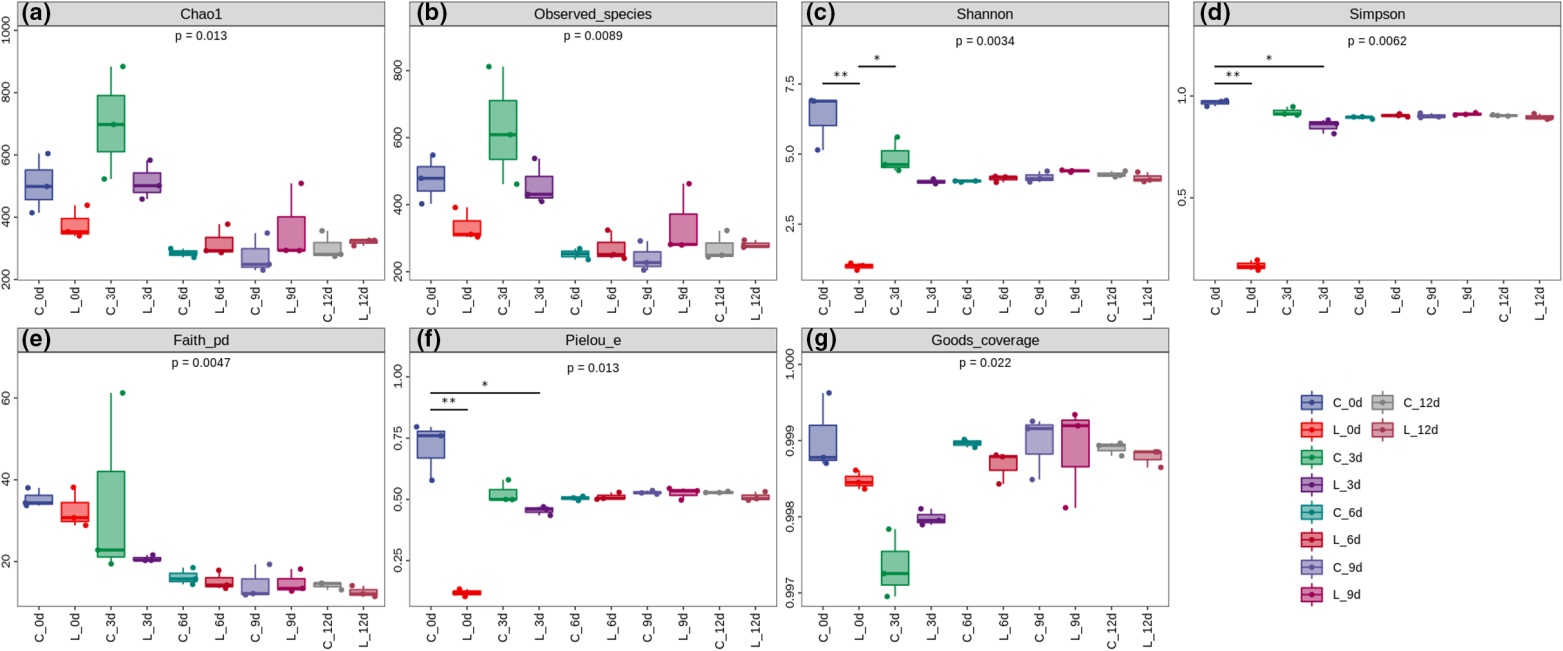
Richness diversity estimator of bacterial communities in overwrapped packaged beef during chilled storage up to 12 days. C ‐ control, without re‐contamination (G0); L ‐ beef samples re‐contaminated by *L. sakei* RS‐25 with the initial number of LAB reached 6.0 log CFU/g (G1)

According to the Good's coverage index, the data coverage is more than 99.7%, indicating that the genetic information of the main microbial community sequence regions in beef has basically been discovered, (Figure 2g Good's coverage). In the control group, the microbial diversity values were higher on the 0 and 3 d of storage, and the diversity index on 3 d was higher than that on 0 d, indicating that a variety of microorganisms showed an increasing trend in the early stage of storage. Diversity and richness of the microbial communities stabilized after 6 days of storage. For the LAB treatment group, the diversity microflora in beef was significantly reduced (Figures 2c Shannon, *p* = .0034; 2D Simpson, *p* = .0062; 2F Pielou's evenness, *p* = .013).

#### 3.6.2 Bacterial composition in fresh beef bacterial composition in fresh beef

**FIGURE 3:**
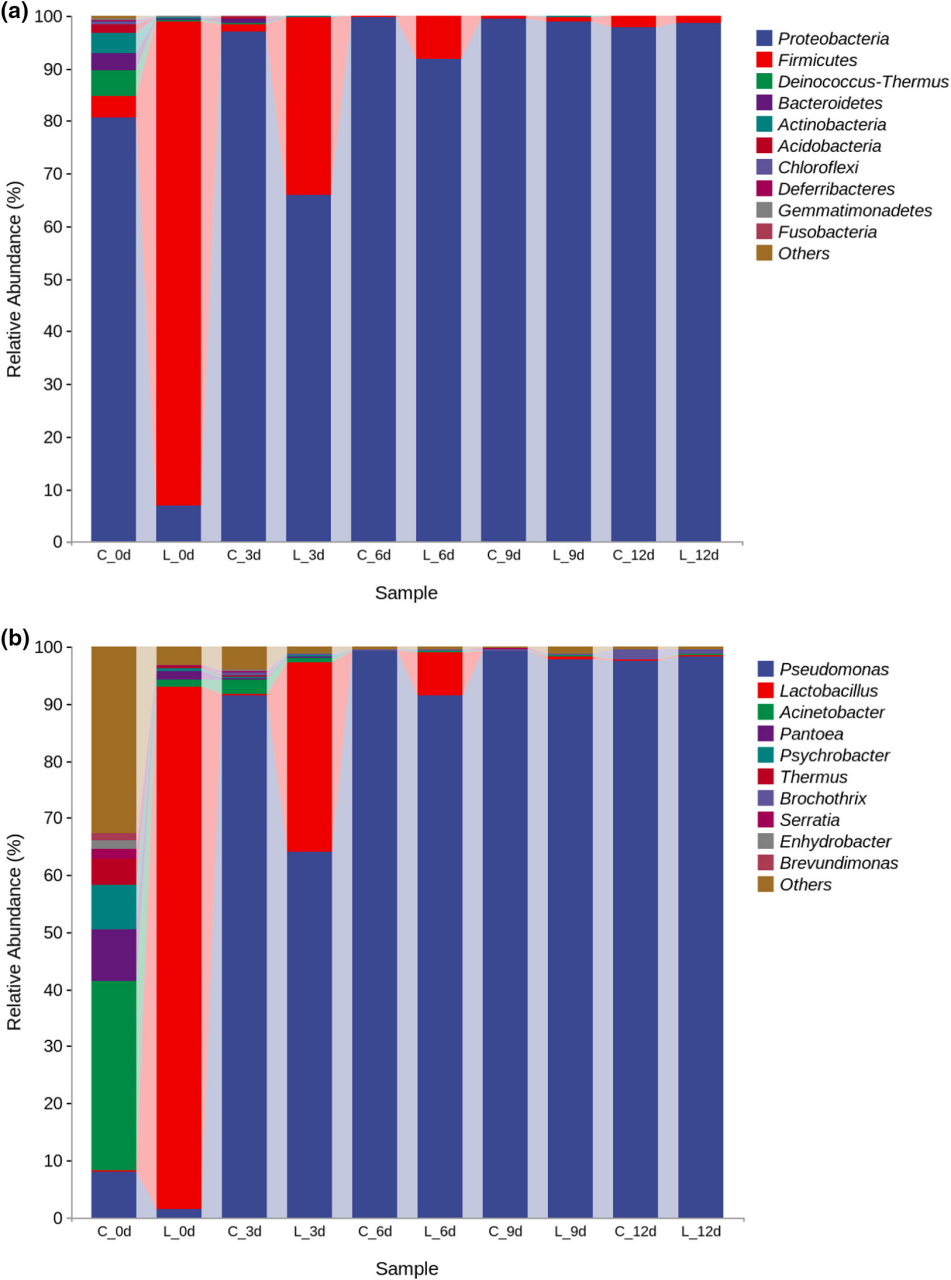
Relative abundance of the bacteria at the phylum level (a) and genus level (b) in overwrapped packaged beef during chilled storage up to 12 days. C ‐ No inoculation treatment (G0); L ‐ beef samples were treated with *L. sakei* RS‐25 at 6 log CFU/g (G1)

Ten dominant bacteria phyla were identified in all beef bacterial DNA samples (Figure 3a). In the control group (G0) with a storage period of 0 day, the main dominant microflora was *Proteobacteria* (80.68%), *Deinococcus‐Thermus* (4.68%), *Firmicutes* (4.19%), *Actinobacteria* (3.74%), *Bacteroidetes* (3.47%) and *Acidobacteria* (1.50%), etc. Then with the extension of storage time, the proportion of *Proteobacteria* increased continuously and reached 99.66% on day 6 of storage. By contrast, after inoculation with *L. sakei*, the treatment group (G1) showed a significantly different microbial composition from the control. *Firmicutes* (92.07%) and *Proteobacteria* (6.93%) were the dominant bacterial phyla in the treatment group on day 0. Subsequently, the relative abundance of *Firmicutes* began to decrease gradually and dropped to 33.8% and 8.1% on the 3 and 6 d of storage, respectively. Furthermore, *Proteobacteria* became the main dominant bacteria phyla after storage for 9 days, accounting for more than 97.9% of the beef microflora in the control and treatment groups.

The changes and relative abundance of all beef microflora samples at the genus level are shown in Figure 3b. *Acinetobacter* spp. (33.32%), *Pantoea* spp. (9.10%), *Pseudomonas* spp. (7.95%), *Psychrobacter* spp. (7.82%), *Thermus* spp. (4.47%), *Serratia* spp. (1.62%), *Enhydrobacter* spp. (1.53%) and *Brevundimonas* spp. (1.37%). were the dominant microflora in beef at 0 d of storage. With the prolonging of storage time, *Pseudomonas* spp. became the dominant microflora rapidly, and its relative abundance reached 91.5% after storage for 3 days. The treatment group (G1) showed a significantly different microflora composition from the control group after inoculation with 6 log CFU/g of *L. sakei*, and the *Lactobacillus* occupied the dominant microflora within 6 days (accounted for 91.7%, 33.3% and 7.74% on 0, 3, and 6 d, respectively). After 6 days of storage, the beef microflora was completely dominated by *Pseudomonas* spp., and these results were consistent with the microbial count results, which proved that the beef had tended to spoil at this time.

Different processing environments affect the initial microflora of beef. Chen et al. (2020) investigated beef bacterial composition from two abattoirs and found that the environment of abattoir B was closer to the beef processing environment in this study, and *Pseudomonas* spp. (31.4%), *Acinetobacter* spp. (17.2%), and *Psychrobacter* spp. (10.1%) were the predominant bacteria. In this study, *Pseudomonas* was the dominant bacteria, although the relative abundance of *Pseudomonas* spp. in the treatment group (G1) was only 1.39%, but this did not affect its later development as a dominant spoilage strain (Figure 3b). Recent work has shown that *Pseudomonas* is more likely to become the dominant spoilage bacteria in oxygen permeable environment (Mansur et al., 2019; Pennacchia et al., 2011; Wang et al., 2018). Spoilage *Pseudomonas* spp., grown on chilled meats, has a range of properties that aid their survival under low temperatures, giving it a competitive advantage. *Pseudomonas* have strong spoilage ability due to a capacity to produce proteolytic and lipolytic enzymes (Remenant et al., 2015; Stanborough et al., 2018). When the glucose and lactic acid in the meat are depleted, it secretes extracellular proteases that break down the connective tissue between muscle fibers, allowing bacteria to penetrate the muscle tissue (Wickramasinghe et al., 2019). Gupta and Nagamohini (1992) study showed that at 37°C, *Pseudomonas* spp. was able to penetrate 3 cm in meat after 24 h, and its penetration in muscle was far greater than that of *Lactobacillus* spp., reflecting the differences in the nature and extent of protease production between *Pseudomonas* and *Lactobacillus*. And, even at low cell numbers, psychrotrophic spoilage *pseudomonads* is capable of secreting cold‐active proteases and lipases, and at temperatures below the optimum growth temperature, it produces more lipases (Wickramasinghe et al., 2019). Both the siderophores' production capacity and efficient utilization rate of glucose make *Pseudomonas* superior in the competition for nutrients (Gram et al., 2002; Tsigarida et al., 2003). In addition, its low oxygen affinity and effective competition for iron make it easier for *Pseudomonas* to dominate meat foods stored aerobically during chilled storage (Ellis et al., 2000; Mohareb et al., 2015). On the contrary, there is only a small amount of glucose and glucose‐6‐phosphate in lean meat (Lücke, 1994), so that LAB do not have enough fermentable sugars, which reduces the lactic acid and other metabolites of LAB and affects its antibacterial effect.

**FIGURE 4:**
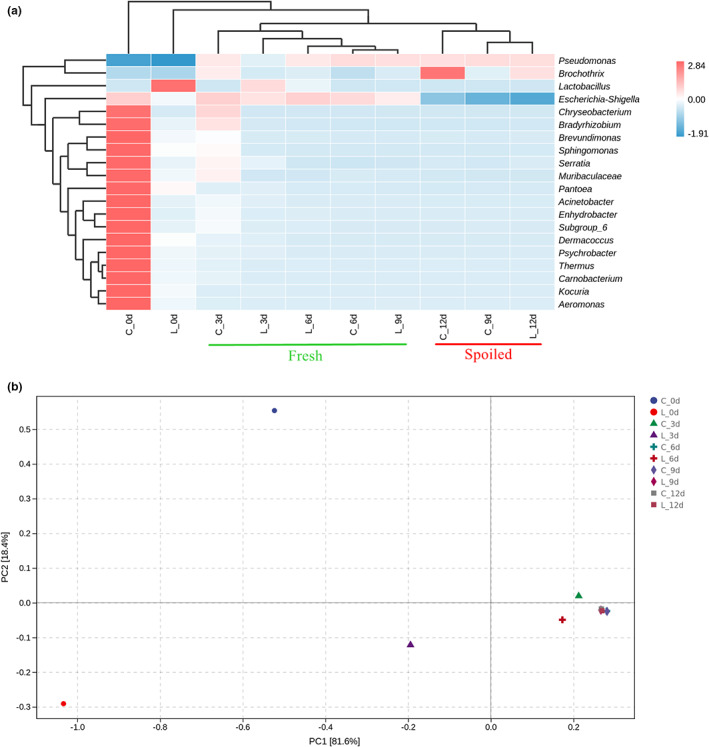
Community heatmap analysis (a) and principal component analysis (PCA; b) at the genus‐level of different microbiota in overwrapped packaged beef during chilled storage up to 12 days. C ‐ No inoculation treatment (G0); L ‐ beef samples were treated with *L. sakei* RS‐25 at 6 log CFU/g (G1).

The heatmap was used to describe the relative abundance changes of the top 20 genera in 12 days, and the cluster tree was drawn above the graph by unweighted pair‐group method with arithmetic (UPGMA) means according to the abundance similarity (Figure 4a). We observed that the control group (G0) had the most abundant microflora at 0 d of the storage period, while the addition of *L. sakei* in the treatment group (G1) significantly reduced the diversity of microflora. Alpha diversity analysis also verified this finding (Figure 2c Shannon; 2D Simpson; 2F Pielou's evenness). It is worth mentioning that after inoculation with *L. sakei*, alpha analysis also showed a decrease in abundance (In other words, the number of ASV/OTU was reduced), which was also consistent with the current results (Figure 2a Chao1; 2B Observed_species). And *L. sakei* RS‐25 also affected the structure of the microflora in beef at the later stage of storage. In cluster analysis, the microflora structure of the treatment group at 9 and 12 d of storage was more similar to that of the control group at 6 and 9 d, respectively (Figure 4a). Taking the 6th day of storage as the time demarcation point, the microbial flora structure could be divided into fresh (C_3d, C_6d, L_3d, L_6d, L_9d) and spoiled (C_9d, C_12d, L_12d). It is worth noting that the microflora structure of the LAB treatment group on the 9th day (L_9d) was more similar to the fresh one. This revealed that the presence of *L. sakei* RS‐25 delayed the changes of microflora structure.

For principal component analysis (PCA), the maximum variations of microbiota in beef samples were 81.6% (PC1) and 18.4% (PC2), representing a strong regional separation (Figure 4b). We clearly observed that control group (G0) on 6 d was clustered with 9 and 12 d of the two groups (G0 and G1), which also illustrated that its microbial composition was more similar. On the contrary, the treatment group on 6 d deviated from the completely spoiled microflora structure. The presence of LAB reduced the diversity and richness of beef microflora and optimized the microflora structure to a certain extent. Chen et al. (2020) also speculated that the dominance of *Lactobacillus* spp. was suggested an important factor affecting shelf life. However, as analyzed above, *L. sakei* did not effectively control the growth of *Pseudomonas* spp. maybe due to the metabolic environmental factors (Morales et al., 2020). Although *L. sakei* did not extend the shelf life of beef in overwrapped packaging, it delayed the succession process of the beef microflora structure to bacteria with high spoilage potential. At last, it should be noted that some *L. sakei* strains can break down amino acids in meat to produce unpleasant flavors. Leisner et al. (1996) reveal that one *L. sakei* 1218 strain developed a distinct sulfide odor after 3 weeks of storage in vacuum‐packed beef and methods should be employed to inhibit the growth of such specific strain. Therefore, to be successful in bio‐conservation, it is necessary to further explore the interspecific diversity of LAB during the storage, especially for the ones who have the ability to decompose fat and protein in meat.

## 4 CONCLUSIONS

This study concluded that the addition of *L. sakei* RS‐25 as a protective culture to fresh beef inhibited the growth of *S*. *typhimurium* and *B. thermosphacta* within 6 days of storage. In addition, RS‐25 had no negative effects on beef pH, TBARS, and TVB‐N, and also delayed the deterioration of meat color. Furthermore, the microbial composition analysis indicated that RS‐25 had an obvious optimization effect on the microbial flora structure of fresh beef, which could delay the evolution trend of microbial flora to spoilage flora. Nevertheless, further studies should consider the practical application of meat substrate, packaging environment, and other factors on the impact of bioprotective bacteria, and to select suitable initial bacteria quantity, or combine with other antibacterial technologies to achieve a better antibacterial effect.

## CONFLICT OF INTEREST

No conflict of interest exits in the submission of this manuscript, and this manuscript has been approved by all authors for publication.

## Data Availability

The data that support the findings of this study are available from the corresponding author, upon reasonable request.
